# Live Birth and Cumulative Live Birth Rates in Expected Poor Ovarian Responders Defined by the Bologna Criteria Following IVF/ICSI Treatment

**DOI:** 10.1371/journal.pone.0119149

**Published:** 2015-03-06

**Authors:** Joyce Chai, Vivian Chi-Yan Lee, Tracy Wing-Yee Yeung, Raymond Wun-Hang Li, Pak-Chung Ho, Ernest Hung-Yu Ng

**Affiliations:** Department of Obstetrics and Gynaecology, University of Hong Kong, Hong Kong Special Administrative Region, China; INRA, FRANCE

## Abstract

**Objective:**

To determine the live birth and cumulative live birth rates of expected poor ovarian responders according to the Bologna criteria and to compare their outcomes with those of expected normal responders

**Design:**

Retrospective analysis

**Setting:**

University infertility clinic

**Patients:**

A total of 1,152 subfertile women undergoing their first in vitro fertilization (IVF) cycle

**Interventions:**

Women were classified into 4 groups according to the Bologna criteria for comparison

**Main Outcome Measure(s):**

Live birth and cumulative live birth rates

**Results:**

Women with expected poor response (POR) had the lowest live birth rate than the other 3 groups (23.8%, p = 0.031). Cumulative live birth rates were significantly lower in those with expected POR than those with expected normal ovarian response (NOR) (35.8% vs 62.8%, p<0.0001). In the subgroup analysis, the cumulative live birth rates in expected PORs were significantly lower in those who had ≤3 oocytes retrieved (18.6% for ≤3 oocytes vs 44.0% for >3 oocytes, p = 0.006) whereas the live birth rates in fresh cycle did not differ (17.8% vs 30.9%, p = 0.108).

**Conclusion:**

Women who were expected POR according to the Bologna criteria had lower live birth and cumulative live birth than expected NOR but they still can achieve reasonable treatment outcomes and IVF treatment should not be precluded.

## Introduction

The success of *in-vitro* fertilization (IVF) depends on an adequate follicle recruitment by using ovarian stimulation with gonadotrophins. Unfortunately early reports suggest that the incidence of poor ovarian response (POR) ranges from 9% to 24% in women undergoing ovarian stimulation for IVF [1]. POR remains one of the main therapeutic challenges in modern reproductive medicine and despite many strategies have been proposed for the management of this particular cohort of women, none is considered to be unequivocally effective [2–3].

One of the limitations in interpreting the relevant literature is the huge discrepancy in the definitions used for POR [4]. The European Society of Human Reproduction and Embryology (ESHRE) made the first attempt in 2011 to reach a consensus on the definition of POR (the Bologna criteria) with the aim to characterize this condition in a simplified and reproducible approach [5]. According to the Bologna criteria, the minimal criteria needed to define POR are the presence of at least two of the following three features: [i] advanced maternal age (≥40 years) or any other risk factors for POR; [ii] a previous POR (≤3 oocytes with a conventional stimulation protocol); and [iii] an abnormal ovarian reserve test. A recent retrospective study reported for the first time an overall low live birth rate of 6% per cycle in Bologna poor responders irrespective of age and treatment protocol used [6].

In a realistic approach, the term POR should refer to the ovarian response and therefore, one stimulated cycle is considered essential for the diagnosis of POR. However, the trend of delaying first pregnancies has led many women to present to their first IVF treatment at an advanced age. Aging results in a physiological decline in the ovarian follicle pool and the prevalence of POR is known to increase with age [5]. Ovarian reserve tests (ORT) help to provide an indirect measure of the primordial follicle pool and among all the tests, antral follicle count (AFC) and anti-Mullerian hormone (AMH) are considered as the most reliable and accurate markers of ovarian reserve and have the best sensitivity and specificity for predicting ovarian response [7–8]. Therefore, women over 40 years of age with an abnormal ORT may be classified as poor responders since both advanced age and an abnormal ORT may indicate reduce ovarian reserve and act as a surrogate of ovarian stimulation cycle. In this case, the women should be more properly defined as expected PORs [5]. The precognition of the pregnancy potential in this group of expected PORs can help clinicians to provide important counseling information to women and avoid unnecessary financial burden and disappointing outcome of IVF treatment. To date no data are available regarding live birth and cumulative live birth rates in expected PORs according to the Bologna criteria.

The purposes of the present study were (i) to explore the live birth and cumulative live birth rates for Bologna expected poor ovarian responders undergoing first cycle of IVF treatment; and (ii) to compare the results with those with expected normal ovarian response and those with poor ovarian reserve (either age ≥40 years or abnormal ovarian reserve test) alone.

## Materials and Methods

This was a retrospective study carried out at the Centre of Assisted Reproduction and Embryology, The University of Hong Kong—Queen Mary Hospital, Hong Kong. Clinical details of all treatment cycles were prospectively entered into a computerized database, which were checked for accuracy and completeness on a regular basis and were retrieved for analysis. Ethical approval was obtained from the Institutional Review Board of the University of Hong Kong/Hospital Authority Hong Kong West Cluster for this retrospective study. Written informed consent was obtained from women for the use of their clinical records.

### Study Population

We analyzed data of subfertile women who underwent first IVF/intracytoplasmic sperm injection (ICSI) cycles between January 2007 and December 2009. Women with incomplete records and cycles carried out for pre-implantation genetic diagnosis or those using donor oocytes were excluded.

Women were divided into four groups for comparison: (i) Expected normal ovarian response (ENOR); (ii) Expected poor ovarian response (EPOR); (iii) Bologna 1; (iv) Bologna 3. ENOR was those aged <40 with normal ORT and no history of ovarian surgery/endometrioma. EPOR was defined according to the Bologna criteria as women with advanced maternal age (≥40 years) or history of ovarian surgery/endometrioma together with an abnormal ORT. Bologna 1 referred to those women aged ≥40 or had history of ovarian surgery/endometrioma but normal ORT while Bologna 3 referred to those aged < 40 and no history of ovarian surgery/endometrioma but had an abnormal ORT.

### Determination of abnormal ORT

The ESHRE consensus group defined abnormal ORT as AFC of <5–7 or AMH of <0.5–1.1 ng/ml [5] based on previous systematic reviews [7, 9]. The ability of AFC and AMH to predict a yield of three oocytes or less (as suggested by the Bologna criteria) with a conventional stimulation protocol (as detailed below) was assessed by receiver operator characteristic (ROC) curve analysis using the data of this particular cohort of eligible women once stimulation was performed. These gave areas under the curve (AUC) values of 0.77 for AFC (95% confidence interval 0.75–0.80, p<0.0001) and 0.80 for AMH (95% confidence interval 0.78–0.83, p<0.0001). The optimum cut-off value for AFC was ≤6 (sensitivity of 69% and specificity of 74%) and for AMH was ≤2 ng/ml (sensitivity of 78% and specificity of 71%), and these values were used to define abnormal ORT in our cohort.

### Ovarian stimulation and embryo transfer

Details of the stimulation cycle have been previously reported [10]. All women were treated either with the long GnRH agonist protocol or the GnRH antagonist protocol for pituitary down-regulation. The initial dose of stimulation was determined according to the baseline AFC (AFC ≥15: 150 IU per day; AFC between 6–14: 300 IU for the first two days followed by 150 IU daily; AFC ≤5: 450 IU for the first two days followed by 225 IU daily). Cycles would be cancelled if no follicular growth was observed after 14 days of maximum stimulation. Human chorionic gonadotrophin was given to trigger the final oocyte maturation when at least one leading follicle reached 18 mm in diameter. Fertilization was carried out *in vitro* either by conventional insemination or ICSI depending on semen parameters. Fresh embryo transfer (ET) was carried out with replacement of at most two embryos of the best quality available two days after retrieval. Excess good quality embryos were also cryopreserved on the day of ET. Pregnancies were confirmed by positive urine hCG tests and transvaginal ultrasonographic evidence of a gestational sac.

### Cryopreservation and frozen-thawed embryo transfer

The details of the freezing and thawing protocols were reported previously [11]. The frozen embryos were thawed on the morning of FET. Embryos were discarded if more than 50% of original blastomeres were lysed or degenerated upon thawing. Frozen-thawed embryos were transferred in natural cycles in ovulatory women, or in either clomiphene-induced or hormone replacement cycles for anovulatory women. A maximum of two frozen embryos were allowed to be transferred in any one FET cycle.

### Collection of clinical information

Clinical information including age, body mass index, basal serum levels of AMH, and AFC were collected. Cycle characteristics such as days of stimulation, total dosage of gonadotrophin, number of oocytes retrieved, and number of transferrable embryos, and cycle cancellation rate due to poor response were recorded.

Clinical pregnancy was defined as the presence of a gestational sac by ultrasonography, whereas miscarriage rate per clinical pregnancy was defined as the proportion of patients who failed to continue development to 20 weeks of gestation in all clinical pregnancies. Pregnancy outcome was collected from all pregnant women by postal questionnaire or by phone. Live birth was defined as the delivery of a fetus with signs of life after 24 completed weeks of gestational age.

### Main outcome measures

The main outcome measures were live birth and one-cycle cumulative live birth rates. Only women who completed replacement of all available frozen embryos were included for analysis of the cumulative live birth rate. In addition, within the group of EPOR, live birth and cumulative live birth rates were analyzed according to the number of oocytes retrieved at the threshold of three (≤3 and >3 oocytes retrieved), since this threshold is one of the Bologna criteria.

### Statistical analysis

For each group of women, categorical data are presented by number of cases and percentages. Continuous data are presented as median with interquartile range. Statistical analysis was performed using Mann-Whitney test, Kruskal-Wallis test or Student’s *t* test, as appropriate. The chi-squared test and Fisher’s exact test were used for the comparisons of categorical variables. Statistical analysis was carried out using the Statistical Program for Social Sciences (SPSS Inc., Version 20.0, Chicago, USA). The two-tailed value of P<0.05 was considered statistically significant.

## Results

A total of 1,156 women underwent the first IVF cycle during the study period and four were excluded because of incomplete data. The prevalence of ENOR and EPOR among them was 48.8% (562/1152) and 13.9% (160/1152) respectively. Eighty-two of them (7.1%) fulfilled Bologna criteria 1 alone and 348 (30.2%) fulfilled Bologna criteria 3 alone.

The baseline characteristics were presented in Table 1. The age and the type of subfertility were comparable among the 4 different groups. AMH and AFC values, as expected, were significantly lower in EPOR women and women with abnormal ORT alone (Bologna 3) when compared with ENOR women. Male factor was the commonest cause of subfertility among the 4 groups, but the prevalence was significantly higher in ENOR group and Bologna 3 group.

**Table 1:** Patients’ baseline characteristics.

	ENOR (N = 562)	EPOR (N = 160)	Bologna 1 (N = 82)	Bologna 3 (N = 348)	P value
Age (years)	34 (32–37)	38 (35–41)	36 (33–40)	37 (34–38)	NS
Body mass index (kg/m^2^)	21.3 (19.6–23.2)	21.0 (19.4–22.7)	20.9 (19.6–23.1)	21.2 (19.6–23.2)	<0.001
AMH (ng/ml)	4.94 (3.50–7.63)	1.05 (0.54–1.70)	3.75 (2.78–5.82)	1.33 (0.87–1.86)	<0.001
AFC	14 (10–18)	5 (3–6)	11 (9–13)	5 (4–7)	<0.001
Duration of subfertility (years)	4 (3–6)	3 (2–5)	4 (3–6)	4 (3–6)	<0.001
Primary subfertility	379 (67.4)	107 (66.9)	56 (68.3)	229 (65.8)	NS
Secondary subfertility	183 (32.6)	53 (33.1)	26 (31.7)	119 (34.2)	NS
Cause of subfertility					<0.001
Male factor	366 (65.1)	63 (39.4)	36 (43.9)	219 (62.9)	
Tuboperitoneal factor	79 (14.1)	25 (15.6)	8 (9.8)	47 (13.5)	
Endometriosis	12 (2.1)	23 (14.4)	11 (13.4)	8 (2.3)	
Unexplained	79 (4.1)	39 (24.4)	20 (24.4)	58 (16.7)	
Anovulation	26 (4.6)	10 (6.2)	7 (8.5)	16 (4.6)	

Data expressed as median (interquartile range) or number (%)

The cycle characteristics and reproductive outcomes were shown in Table 2. Women who were EPOR required significantly longer duration of stimulation and higher dosage of gonadotrophin, and they had significantly lower number of oocytes retrieved and number of transferrable embryos. Only one patient had cycle cancellation. The median number of oocytes retrieved in EPOR group was 5 and 31.9% of them had 3 or less oocytes retrieved. The live birth rates per started cycle were 35.9% and 23.8% for ENOR and EPOR respectively (p = 0.004). For those fulfilling Bologna 1 or Bologna 3 alone, the live birth rates per started cycle were comparable with ENOR (36.6% in Bologna 1 vs 35.9% in ENOR, p = 0.90; 32.5% in Bologna 3 vs 35.9% in ENOR, p = 0.31) but significantly higher than EPOR (36.6% in Bologna 1 vs 23.8% in EPOR, p = 0.05; 32.5% in Bologna 3 vs 23.8% in EPOR, p = 0.05).

**Table 2:** Cycle characteristics and reproductive outcomes.

	ENOR (N = 562)	EPOR (N = 160)	Bologna 1 (N = 82)	Bologna 3 (N = 348)	P value
Duration of stimulation (days)	11 (9–12)	11 (10–13)	11 (9–12)	12 (10–13)	<0.001
Total dose of gonadotrophin (IU)	1800 (1500–2400)	3150 (2700–3600)	1950 (1650–2700)	2850 (2438–3375)	<0.001
Peak E_2_ (pmol/L)	12343 (8375–18388)	5682 (4145–10262)	11508 (8061–17164)	7279 (4229–11334)	<0.001
Number of eggs retrieved	11 (8–15)	5 (3–8)	11 (8–13)	6 (4–9)	<0.001
Number of transferrable embryos	5 (3–8)	2 (1–4)	5 (2–7)	3 (2–5)	<0.001
Cycle cancellation due to poor response	0 (0)	1 (0.6)	0 (0)	1 (0.3)	NS
Patients with ≤3 oocytes retrieved	26 (4.6)	51 (31.9)	2 (2.4)	78 (22.4)	<0.001
Clinical pregnancy	259 (46.1)	53 (33.1)	37 (45.1)	144 (41.4)	0.02^a^
Miscarriage	57/259 (22.0)	15/53 (28.3)	7/37 (18.9)	31/144 (21.5)	NS
Live birth	202 (35.9)	38 (23.8)	30 (36.6)	113 (32.5)	0.031^b^
Cumulative live birth	323/514 (62.8)	48/134 (35.8)	41/76 (53.9)	146/314 (46.5)	<0.0001^c^

Data expressed as median (interquartile range), n(%) or n/total (%)

^a^Clinical pregnancy rate was significantly lower in EPOR than ENOR group

^b^Live birth rate was significantly lower in EPOR than the other 3 groups

^c^Cumulative live birth rate was significantly lower in EPOR than the other 3 groups. Bologna 3 group had significantly lower cumulative live birth rate than ENOR.

Overall, 1,038 women completed replacement of all available frozen embryos. The cumulative live birth rate of EPOR group was significantly lower than ENOR group (35.8% vs 62.8%, p<0.0001). Those fulfilling Bologna 1 had similar cumulative live birth rate to ENOR (53.9% vs 62.8%, p = 0.16) but higher than EPOR (p = 0.01). Those fulfilling Bologna 3 had significantly lower cumulative live birth rate than ENOR (46.5% vs 62.8%, p<0.0001) but higher than EPOR (p = 0.04).

In the EPOR group, 142 out of 160 women had fresh embryo transfer and 134 had completed transfer of all available embryos. Further analysis according to ovarian response showed that the live birth rates in EPOR did not significantly differ between poor response (≤3 oocytes retrieved) and normal response (>3 oocytes retrieved) as shown in Table 3. However, for those with have >3 oocytes retrieved, the cumulative live birth rate was significantly higher (44.0% vs 18.6%, p = 0.006). When comparing the 134 who had completed transfer of all available embryos, women aged <40 with history of ovarian surgery/endometrioma and abnormal ORT performed better than those with advanced age ≥40 and abnormal ORT in terms of cumulative live birth rates (46.4% (39/84) vs 18.0% (9/50), p<0.001). In Bologna 3 the live birth rates also did not significantly differ between poor response and normal response (36.1% vs 34.9%), but for those with have >3 oocytes retrieved, the cumulative live birth rate was significantly higher (49.4% vs 34.9%, p = 0.048) (Table 4).

**Table 3:** Reproductive outcomes in EPOR by ovarian response.

	> 3 oocytes retrieved	≤ 3 oocytes retrieved	P value
Live birth in fresh cycle	30/97 (30.9%)	8/45 (17.8%)	NS
Cumulative live birth	40/91 (44.0%)	8/43 (18.6%)	0.006

**Table 4:** Reproductive outcomes in subjects fulfilling Bologna criteria 3 only stratified by ovarian response.

	> 3 oocytes retrieved	≤ 3 oocytes retrieved	P value
Live birth in fresh cycle	91/252 (36.1%)	22/63 (34.9%)	NS
Cumulative live birth	124/251 (49.4%)	22/63 (34.9%)	0.048

Multivariable logistic regression analysis revealed that age and number of embryos replaced were significantly associated with the live birth rate with OR 0.87 (95% CI 0.83–0.92) and OR 4.13 (95% CI 2.05–8.28) respectively, after adjusting for other characteristics including EPOR/ENOR classification, AFC, AMH, and body mass index. Likewise, age and total number of transferrable embryos were significant independent predictors of cumulative live birth rates with OR 0.87 (95% CI 0.82–0.92) and OR 1.32 (1.23–1.41) respectively.

## Discussion

The aim of this study was to examine the implications of expected poor ovarian response as defined by the Bologna criteria for women undergoing their first IVF/ICSI treatment, in particular live birth and cumulative live birth rates, which are the most relevant information for women and their clinicians. Our results showed that EPOR were not uncommon among women pursuing IVF/ICSI treatment (13.9%) and it highlighted the poorer prognosis of EPOR in terms of reproductive outcomes when compared to ENOR, but nonetheless they achieved live birth and cumulative live birth rates of 23.8% and 35.8% respectively. Multivariate logistic regression model revealed that EPOR/ENOR was not a significant predictor of live birth or cumulative live birth after adjusting for age, AFC, AMH, body mass index and number of embryos transferred in the fresh cycle / total number of transferrable embryos. Only age and number of embryos were significant predictors of such outcome.

The prevalence of poor ovarian responders varies depending on the definitions of poor response. In general, poor ovarian responders (who did not meet the Bologna criteria) are reported to have low pregnancy rate and low live birth rate [12–13]. The ESHRE group made the attempt to standardize the definition of poor responders in 2011 and subsequent studies have consistently showed low live birth rates of 7.4–9.9% following natural cycle IVF and stimulated IVF in Bologna poor responders [6, 14]. Another study has demonstrated cumulative live birth rate of 12.7%–20.5% after three cycles of ovarian stimulation IVF [15]. Our live birth and cumulative live birth rates, in contrast to previous studies, were more optimistic at 23.8% and 35.8% respectively. This discrepancy is likely attributed to the difference in selection of patients as all the previous studies included women with one or more previous cycles with POR whereas we only included expected POR based on their age/risk factors for POR and results of ovarian reserve test. Women with previous POR to ovarian stimulation are more likely to have a recurrence of a poor response [16], and the pregnancy rate is decreased in the subsequent cycles if abnormal ORTs are present [17].

The median number of oocytes retrieved in Polyzos’s retrospective study [6] was 3 as compared to 5 in our study. Pregnancy prospects reduce when fewer oocytes are retrieved [18] and an increase in the number of oocytes is an independent variable related to live birth rates [6]. Despite only 31.9% of EPOR women had ≤3 oocytes retrieved, our results were consistent in showing that these women had a poorer prognosis in terms of cumulative live birth rates than those who have >3 oocytes retrieved. This confirms that the threshold of 3 oocytes adopted by the ESHRE consensus is adequate to identify women with the poorer prognosis in terms of cumulative live birth rates. Interestingly, within the EPOR women, those with advanced age together with abnormal ORT did poorer than those aged <40 with risk factor for POR and abnormal ORT, reflecting age alone remains the best marker of oocyte quality and the best single predictor of ongoing pregnancy.

The risk of POR generally increases with age [5] and in women with endometriomas with or without cystectomies [19], but their ovarian reserve may still vary and are better assessed by ORTs. Our data suggested that women with Bologna 1 alone had significantly better ovarian response and reproductive outcomes compared with expected POR as defined by the Bologna criteria, possibly related to the better ovarian reserve as reflected by the significantly higher AFC and AMH level. Bologna 1 alone is the less strong in the prediction of POR as only 2.4% of women with Bologna 1 were actually poor responders with ≤3 oocytes retrieved. However, our women with Bologna 3, albeit similar ovarian reserve to EPOR women, achieved significantly higher live birth and cumulative live birth. This is in concordance with our previous study which showed women aged less than 40 with extremely low levels of AMH could still achieve a reasonable live birth rate [20]. Only 22.4% of women with Bologna 3 alone had actually a poor response, and of those with poor response their live birth rates were actually similar to those with better response. Our results highlighted that a single criterion is insufficiently accurate to identify those with poor prognosis, and even new markers like AMH used alone still fail to adequately identify women with the highest probability of being a real POR.

Our study is limited by its retrospective design and therefore there were several baseline characteristics that significantly differed among the groups. In the study design we defined Bologna 1 based on the women’s age, presence of endometrioma and ovarian cystectomy, as these were the easily identifiable risk factors. However, our study failed to address other risk factors for POR including any genetic or acquire conditions possibly linked to a reduced ovarian reserve, as criticized by some authors [21]. It is possible to suspect that an even lower (cumulative) live birth rates in EPOR women who had other identified risk factors. The limitations of AMH assays, particularly with regard to assay bias, should also be taken into account. The AMH cut-off that we used in this study was higher than that suggested by the original Bologna consensus paper [5]. This could be due to the different AMH assays used: the Gen II ELISA kit by Beckman-Coulter was used in this study whereas older assay methods were used in most previous publications reviewed by the Bologna consensus paper [5]. The initial dose of stimulation was determined according to the baseline AFC alone as per department protocol and AMH levels were not taken into account. This could potentially introduce bias as AMH was used as one of the criteria of ORT in classifying women. In addition, our results may not be applicable to other IVF centers as treatment protocols can be different and performance of women can vary among centers. We had a lower cycle cancellation rate of 0.002% (2/1,152) when compared to other studies [22], likely related to our lower threshold for cycle cancellation as we only cancelled the cycle when there was no response whereas other centers usually cancelled the cycle when <3 follicles of size 18 mm were recruited in response to gonadotrophins.

Despite using the Bologna criteria as suggested by the ESHRE group which aimed to establish ‘minimal criteria’ to select a homogeneous population of women in terms of oocytes quantity, our results in terms of reproductive outcomes were significantly different from Polyzos group due to the reasons stated above [6]. They demonstrated a very low live birth rates in Bologna poor responders and suggested some of the women fulfilling the criteria should be considered as potential candidates for oocyte donation programmes. Our results, in contrary, showed reasonable live birth and cumulative live birth rates in expected POR and these women should not be precluded from IVF programmes. This observed difference reinforced the fact that reproductive outcomes can vary widely depending on the criterions used, and the applicability of these criteria is subjected for reevaluation in the future [4]. Further prospective researches are required to determine the actual reproductive potential of Bologna poor responders in IVF treatment, preferably with reference to the criterions used. Nonetheless our results provide valuable information to women who anticipate their first IVF treatment and this is of paramount importance during pre-treatment counseling.

In conclusion, our retrospective data, for the first time, suggested that women who were expected POR according to the Bologna criteria had poorer prognosis in terms of (cumulative) live birth than expected NOR but they still can achieve reasonable treatment outcomes and should not be precluded from attempting IVF.
