# Correction: Live Birth and Cumulative Live Birth Rates in Expected Poor Ovarian Responders Defined by the Bologna Criteria Following IVF/ICSI Treatment

**DOI:** 10.1371/journal.pone.0131334

**Published:** 2015-06-22

**Authors:** Joyce Chai, Vivian Chi-Yan Lee, Tracy Wing-Yee Yeung, Hang Wun Raymond Li, Pak-Chung Ho, Ernest Hung-Yu Ng

The fourth author’s name is spelled incorrectly. The correct name is: Hang Wun Raymond Li. The correct citation is: Chai J, Lee VC-Y, Yeung TW-Y, Li HWR, Ho P-C, Ng EH-Y (2015) Live Birth and Cumulative Live Birth Rates in Expected Poor Ovarian Responders Defined by the Bologna Criteria Following IVF/ICSI Treatment. PLoS ONE 10(3): e0119149. doi:10.1371/journal.pone.0119149
